# The Metabolic Enzyme ManA Reveals a Link between Cell Wall Integrity and Chromosome Morphology

**DOI:** 10.1371/journal.pgen.1001119

**Published:** 2010-09-16

**Authors:** Maya Elbaz, Sigal Ben-Yehuda

**Affiliations:** Department of Microbiology and Molecular Genetics, Institute for Medical Research, Israel-Canada (IMRIC), The Hebrew University-Hadassah Medical School, The Hebrew University of Jerusalem, Jerusalem, Israel; Agency for Science, Technology and Research, Singapore

## Abstract

Synchronizing cell growth, division and DNA replication is an essential property of all living cells. Accurate coordination of these cellular events is especially crucial for bacteria, which can grow rapidly and undergo multifork replication. Here we show that the metabolic protein ManA, which is a component of mannose phosphotransferase system, participates in cell wall construction of the rod shaped bacterium *Bacillus subtilis*. When growing rapidly, cells lacking ManA exhibit aberrant cell wall architecture, polyploidy and abnormal chromosome morphologies. We demonstrate that these cellular defects are derived from the role played by ManA in cell wall formation. Furthermore, we show that ManA is required for maintaining the proper carbohydrate composition of the cell wall, particularly of teichoic acid constituents. This perturbed cell wall synthesis causes asynchrony between cell wall elongation, division and nucleoid segregation.

## Author Summary

The bacterial cell is resistant to extremes of osmotic pressure and protected against mechanical damages by the existence of a rigid outer shell defined as the cell wall. The strength of the cell wall is achieved by the presence of long glycan strands cross-linked by peptide side bridges. The cell wall is a dynamic structure continuously being synthesized and modified to allow for cell growth and division. Damaging the cell wall leads to abnormal cellular morphologies and cell lysis thereby making it a key target for antibiotics. Here we describe the involvement of ManA enzyme in cell wall construction of the rod shaped bacterium *Bacillus subtilis*. In cells lacking ManA the normal extension of the cell wall is blocked. Consequently, *manA* mutant cells display abnormal morphologies and fail to properly package and segregate their DNA resulting in the formation of large cells containing disorganized multiple chromosomal copies. Based on these findings, we propose that appropriate cell wall synthesis is necessary for synchronizing chromosome architecture with cell growth.

## Introduction

The bacterial cell wall is a key determinant of cellular morphology that provides structural support and mechanical protection. The structural dynamics of the bacterial cell wall enable elasticity, growth and division. The cell wall of the Gram positive bacterium *Bacillus subtilis* (*B. subtilis*) is primarily composed of peptidoglycan (PG), a net-like polymer of glycan strands cross-linked by peptide bridges, and anionic phosphate-rich polymers. Both PG and anionic polymers play critical roles in maintaining the structural integrity and viability of the bacterial cell [1].

PG strands comprise alternating units of N-acetylglucosamine (GlcNAc) and N-acetylmuramic acid (MurNAc). PG synthesis initiates in the cytoplasm with fructose-6-phosphate and proceeds through a linear pathway to generate the precursors UDP-GlcNAc and UDP-MurNAc-pentapeptide [2]. The following steps are membrane bound and carried out using a special recycled lipid carrier, undecaprenyl phosphate. Initially, the MraY transferase attaches MurNAc-pentapeptide to the lipid carrier thereby yielding lipid I. Consequently, GlcNAc is added to lipid I by the MurG enzyme producing lipid II. Lipid II carries the basic PG monomer composed of GlcNAc and MurNAc-pentapeptide. Next, lipid II is flipped across the membrane, the PG monomer is cleaved of the undecaprenyl phosphate and incorporated into the growing chain [2]–[4].

Labeling of the newly synthesized PG revealed that it is inserted in a helical pattern along the lateral cell wall [5]–[8]. Accordingly, atomic force microscopy of the *B. subtilis* cell wall exposed helical PG cabling arrangement with glycan strands up to 5 µm in length, longer than the bacterium cell itself [9]. A protein implicated to play a key role in inserting new PG is the actin homolog MreB. MreB forms a dynamic helical scaffold that serves as a platform onto which the cell wall machinery localizes [5], [10]–[16]. *B. subtilis* has three MreB isoforms, called MreB, Mbl and MreBH, which have been demonstrated to colocalize in a single helical structure [17]. Mutations within the genes encoding these isoforms, as well as in other essential PG components, induce severe morphological defects (e.g.: [6], [13], [15], [18]–[21]).

In *B. subtilis* the anionic polymers can be either bound to PG, wall teichoic acid (WTA), or anchored to the cytoplasmic membrane, lipoteichoic acid (LTA). The major form of WTA comprises glycerol phosphate polymer [1], [22]–[24] and the minor form is a polymer of glucose (Glc) and N-acetylgalactosamine (GalNAc) [1], [22], [25], [26]. Fluorescence analysis has revealed that WTA enzymes are localized at division sites and along the lateral sides of the bacterial cells [27]. Similar to the PG pathway, WTA biosynthesis begins with formation of nucleotide sugars in the cytoplasm and proceeds with a membrane step that utilizes the same lipid carrier undecaprenyl phosphate. Notably, mutations in the WTA and/or LTA pathways lead to loss of rod shape and non-uniform thickening of the PG layer [28]–[31], suggesting coordinated biogenesis of the cell wall components.

Here we show that the sugar metabolic enzyme ManA (mannose phosphate isomerase), which is part of the mannose phosphotransferase system, is unexpectedly necessary in rich medium, when mannose is not utilized as a carbon source. In the absence of ManA, cells display abnormal morphologies and fail to properly package and segregate their chromosomes. Furthermore, we demonstrate that these abnormal phenotypes are due to a role played by ManA in cell wall construction. We show that the lack of ManA perturbs proper cell wall carbohydrate composition and thereby causes asynchrony between cell growth, division and nucleoid segregation.

## Results

### ManA is required for cell shape maintenance and proper chromosome segregation in *B. subtilis*

**Figure 1:**
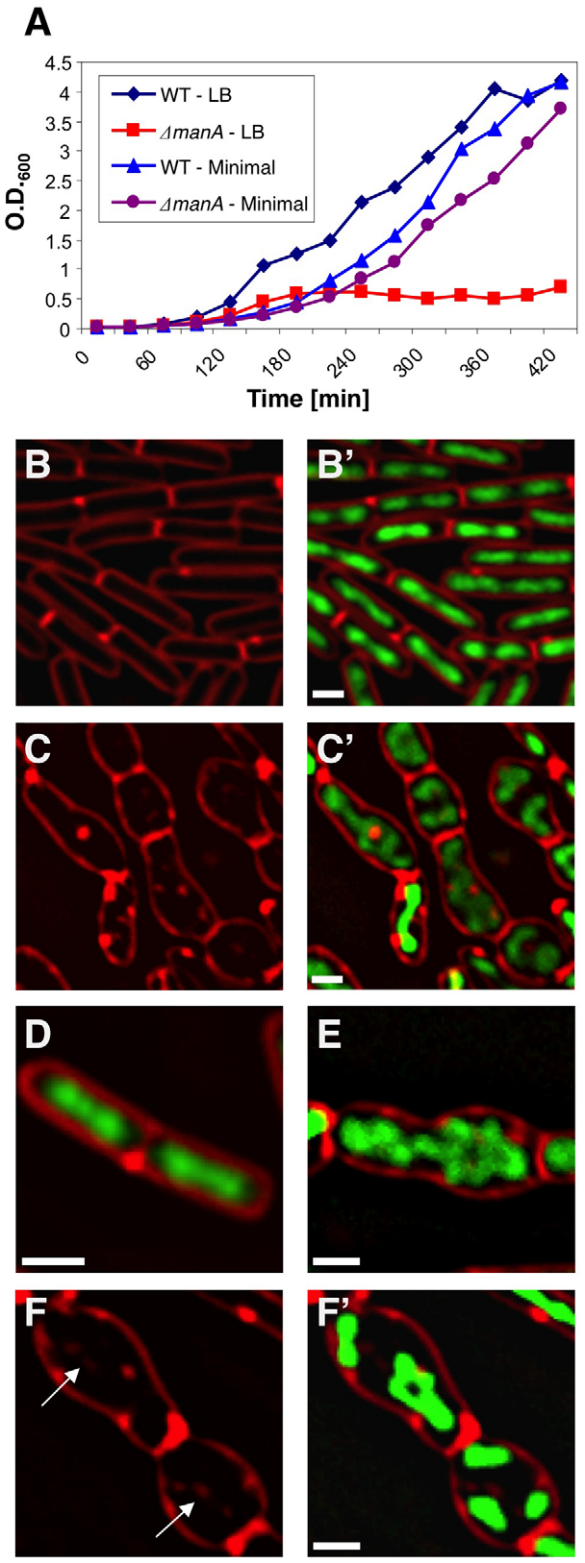
ManA is required for rod shape maintenance and proper chromosome segregation. (**A**) Growth curves of wild type (PY79) and Δ*manA* (ME37) strains grown in rich LB medium (LB) or minimal S7 medium (minimal). (**B**–**F**) Fluorescence microscopy was carried out on wild type (PY79) and Δ*manA* (ME37) cells grown in rich LB medium. DNA and membrane were visualized with DAPI (green) and FM1-43 (red), respectively. (**B**–**C**) Fluorescence images of typical wild type (B, B') and Δ*manA* (C, C') cells. (**D**–**F**) Enlargements of fluorescence images of wild type (D) and Δ*manA* (E, F, F') cells. Arrows in (F) indicate internal membrane invaginations. Scale bars correspond to 1 µm.

To identify new components required for cell division and chromosome segregation, we performed a transposon mutagenesis and screened for *B. subtilis* mutants exhibiting growth defects (Materials and Methods). The selected mutants were then subjected to a visual microscopy assay. One of the slow growing mutants had a striking phenotype with the cells exhibiting a severe shape defect and atypical nucleoid morphologies (Figure 1). Evidently, mutated cells lost the characteristic rod shape typical of wild type *B. subtilis* cells and instead appeared as elongated spheres, which were significantly larger than normal. This spheroid like morphology resembles the phenotype described for mutants defective in cell wall synthesis (e.g.: [6], [13], [15], [18], [20], [21], [29], [30]). In addition, we observed internal membrane invaginations in many of the mutant cells indicative of inappropriate cell divisions (Figure 1F). Moreover, DAPI staining revealed a variety of abnormal nucleoid structures, which have lost their spatial organization in comparison to the well-organized wild type chromosomes (Figure 1).

Cloning and sequence analyses revealed that the transposon was inserted within the coding region of the *manA* (mannose phosphate isomerase) disrupting its function. Accordingly, deletion of *manA* was sufficient to confer the observed defects and ectopic expression of *manA* fully complemented the mutant phenotype (Figure S1A-S1C). *manA* encodes a conserved enzyme that catalyzes the reversible isomerization of fructose-6-phosphate (Fru-6-P) and mannose-6-phosphate (Man-6-P) [32]. Introducing point mutations into the predictable ManA active site abolished the ability of the protein to complement the null *manA* phenotype (Materials and Methods; Figure S1D and S1E). Surprisingly, the *manA* mutant phenotype was displayed by cells grown in rich LB medium when mannose is not exploited as a carbon source. Nevertheless, ManA was found to be produced at significant levels under such conditions (Figure S2). Notably, the *B. subtilis* genome contains a homologue of *manA*, named *pmi* (56% identity), which encodes a second mannose phosphate isomerase. However, a strain bearing a knock out of *pmi* had no observable phenotype and unlike ManA, Pmi was undetectable in rich LB medium (Figure S2), suggesting that the two homologues have non-identical roles. Thus, besides its traditional role as a metabolic enzyme, ManA possesses an additional, previously unrevealed, crucial cellular activity that relies on its enzymatic activity.

### Δ*manA* cells contain multiple copies of fully replicated chromosomes

**Figure 2:**
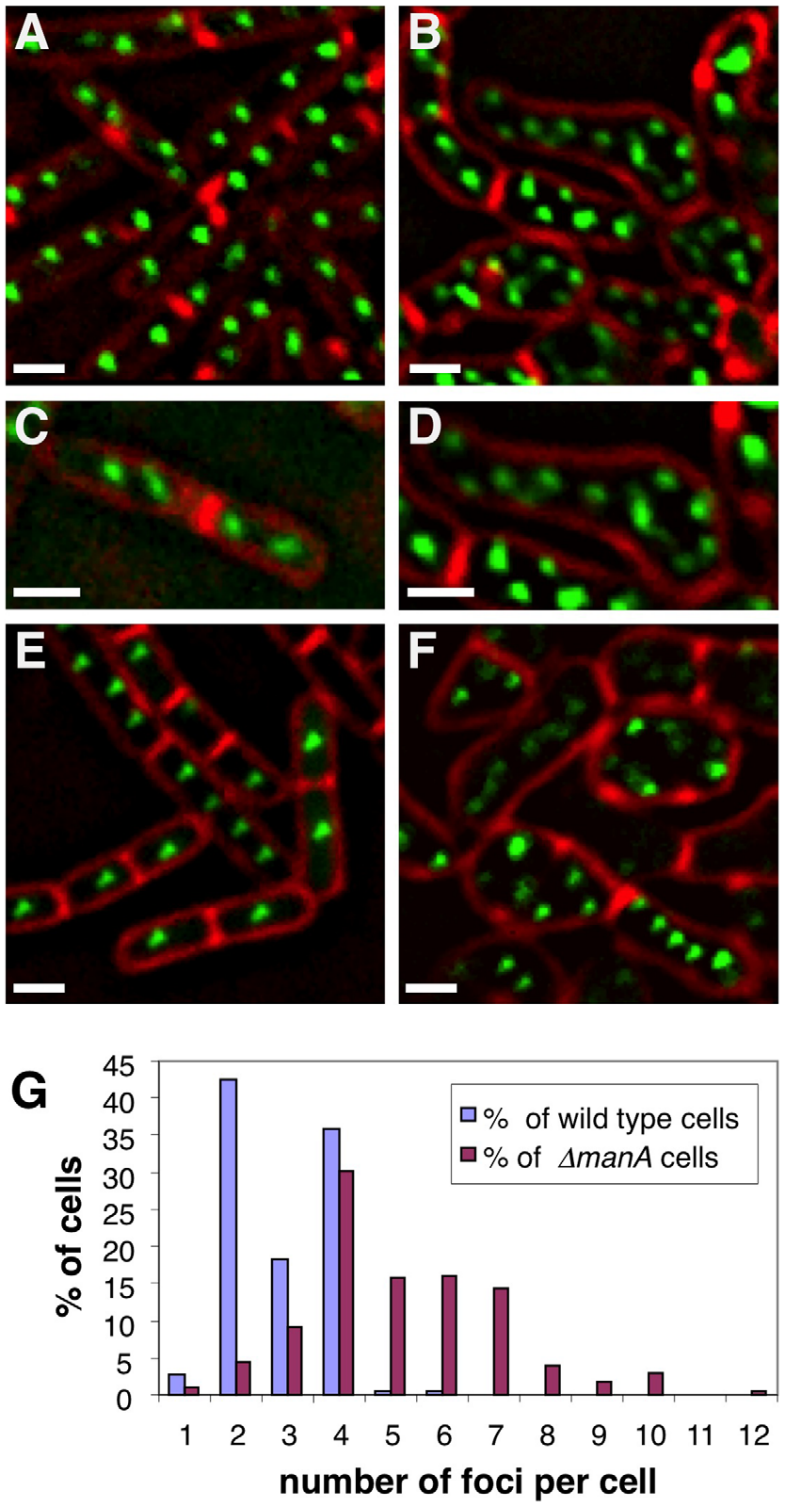
Δ*manA* cells contain multiple copies of fully replicated chromosomes. Origin and terminus regions were visualized in wild type and Δ*manA* cells grown in rich LB medium. (**A**–**B**) Fluorescence images of typical wild type (SB294) (A) and Δ*manA* (ME34) (B) cells producing Spo0J-GFP (green). Cells were stained with the membrane dye FM4-64 (red). (**C**–**D**) Enlargements of fluorescence images of wild type (C) and Δ*manA* (D) cells as in (A–B). (**E**–**F**) Fluorescence images of wild type (ME79) (E) and Δ*manA* (ME82) (F) cells bearing repeated *tetO* units inserted in proximity to the terminus region and producing TetR-GFP (green). Cells were stained with the membrane dye FM4-64 (red). (**G**) Quantification of Spo0J-GFP foci per cell in wild type (SB294) and Δ*manA* (ME34) cells. At least 230 cells were analyzed for each strain. Scale bars correspond to 1 µm.

The observation that Δ*manA* cells display altered nucleoid morphologies suggested that this mutant is perturbed in organizing and segregating the chromosomes. To examine in more detail the nature of Δ*manA* nucleoids, we visualized the replication origin region using GFP fused to Spo0J, a protein that binds near the origins [33]–[35]. Wild type cells producing Spo0J-GFP were observed to contain 2–4 fluorescent foci (Figure 2A and 2C), while the number of origins within the larger Δ*manA* cells frequently exceeded 4 (Figure 2B). Figure 2D exemplifies a characteristic Δ*manA* cell containing as many as 12 copies of origin. Quantification analysis of the number of Spo0J-GFP foci per cell showed that the wild type population exhibited a narrow distribution with more than 99% of the cells containing 1–4 foci, whereas the number of foci in the mutant cells varied widely from 1 to 12, with the majority of cells (55%) bearing more than 4 foci per cell (Figure 2G).

The increased number of origin foci observed within Δ*manA* cells raised two possibilities: either the cells are polyploids containing multiple chromosomal copies, or alternatively these mutant cells erroneously reinitiate replication without the actual completion of chromosome synthesis. To distinguish between these possibilities, we visualized the terminus, which is the last chromosomal region to be replicated. We generated wild type and Δ*manA* strains that harbor TetR–GFP and carry repeated *tetO* units inserted in proximity to the terminus region [36]. Consistent with the idea that Δ*manA* cells are polyploids, the Δ*manA* strain exhibited more TetR–GFP foci than the typical 1–2 foci observed in the wild type strain (Figure 2E and 2F). Nevertheless, to verify that the multiple copies of origin and terminus represent whole chromosomes, we applied DNA microarray to compare the DNA content of wild type and Δ*manA* cells [37] (Materials and Methods). Equal amounts of genomic DNA extracted from wild type and mutant cells were labeled and hybridized to a *B. subtilis* DNA chip. This analysis indicated no significant difference between the two samples implying that amplification of specific DNA regions is not a feature of Δ*manA* cells. Thus, Δ*manA* cells most likely contain several copies of fully replicated chromosomes within each cell.

### Δ*manA* phenotype can be suppressed partially by slowing growth and replication

**Figure 3:**
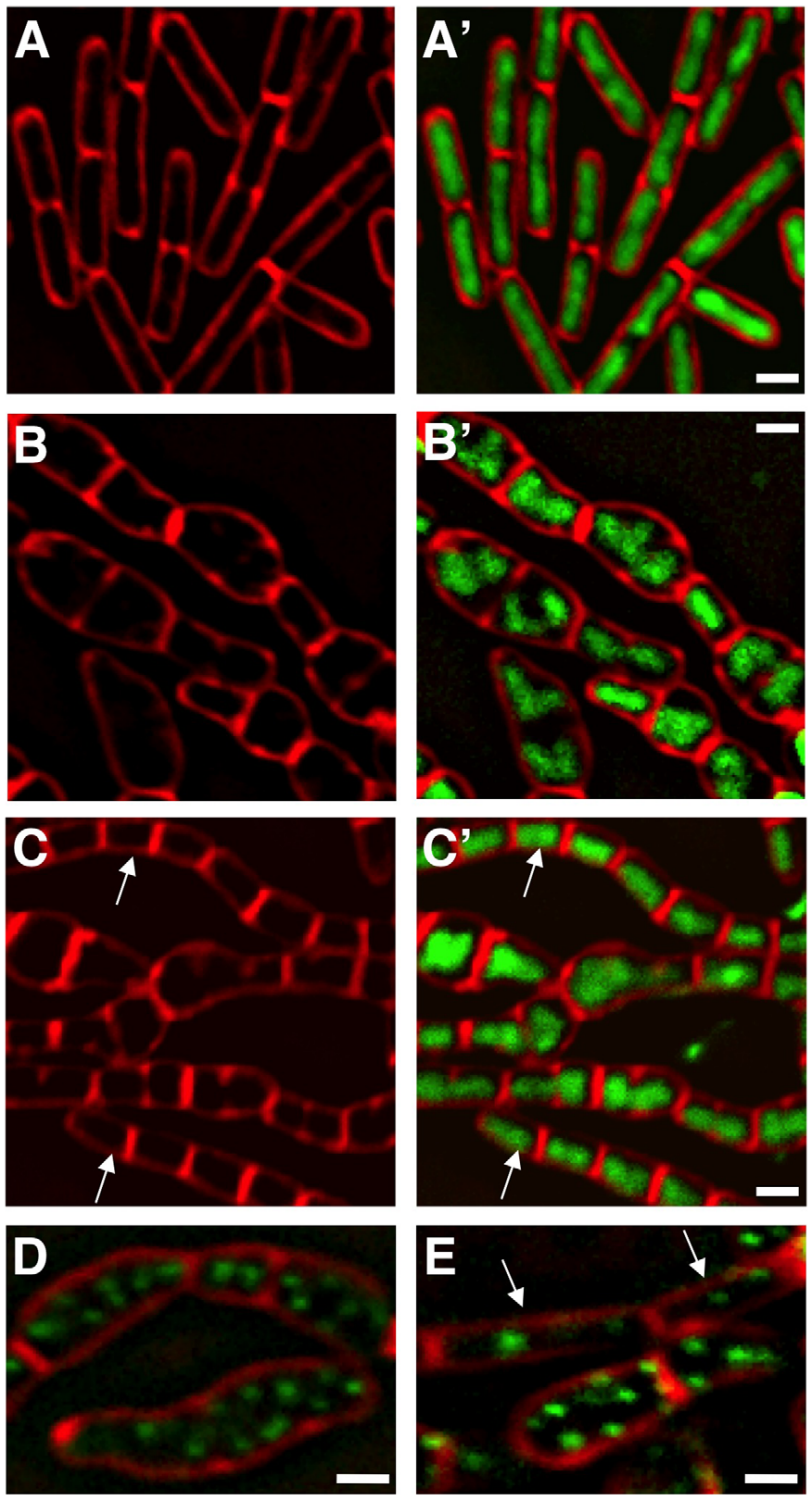
Δ*manA* phenotype is partially suppressed by slowing growth and replication. (**A**) Δ*manA* (ME37) cells grown in S7 minimal medium at 37°C were stained with DAPI (green) and FM1-43 (red) and visualized by fluorescence microscopy. (**B**–**C**) Δ*manA* (ME37) (B, B') and Δ*manA dnaBts* (ME46) (C, C') cells grown at the restrictive temperature (42°C) in rich LB medium were stained with DAPI (green) and FM1-43 (red) and visualized by fluorescence microscopy. (**D**–**E**) Δ*manA* (ME34) (D) and Δ*manA dnaBts* (ME133) (E) cells producing Spo0J-GFP (green) were stained with the membrane dye FM4-64 (red) and visualized by fluorescence microscopy. Arrows in (C, C') and (E) indicate cells with “wild type-like” phenotypes. Scale bars correspond to 1 µm.

Further examination of the Δ*manA* mutant strain revealed that the defects in cell shape and chromosome morphology are growth rate dependent. When growth was slowed, either by reducing the temperature or by growing Δ*manA* cells in minimal medium, an almost normal phenotype was observed (Figure 3A; Figure S3). Accordingly, the levels of ManA production were significantly higher in rich medium (Figure S3). A prominent feature of rapid bacterial growth is the ability to perform multifork replication but still allocate the daughter chromosomes properly into progeny cells [38]. In light of the growth dependence of the Δ*manA* phenotype, we speculated that Δ*manA* cells are defective specifically in performing this complex task and as a consequence become polyploids. In order to test this premise, we investigated whether reducing the occurrence of multifork replication suppresses the Δ*manA* phenotype. To that end, we combined the Δ*manA* allele with a temperature sensitive allele of the replication initiation factor *dnaB* (*dnaBts*) [39], [40]. Incubating this strain at the restrictive temperature leads to inactivation of DnaB and therefore reduces the rate of multifork replication. In line with our expectation, when the Δ*manA dnaBts* strain was grown at the restrictive temperature (42°C) the Δ*manA* phenotype was partially suppressed, as manifested by the frequent appearance of almost wild type cell chains (Figure 3C). Importantly, these “wild-type like” cells were absent when the Δ*manA dnaBts* strain was incubated at the permissive temperature (37°C), or when Δ*manA* strain was incubated at the restrictive temperature (Figure 3B). Notably, by the time of transfer to the restrictive temperature the majority of the cells already acquired the Δ*manA* phenotype and were polyploid, a phenotype which at some point may become irreversible. This occurrence may account for the partial nature of the suppression. To confirm that the appearance of “wild-type like” Δ*manA dnaBts* cells correlates with reduced chromosomal copy number, we visualized the number of replication origins in the Δ*manA dnaBts* strain by expressing Spo0J-GFP. Indeed, the number of Spo0J-GFP foci observed within the suppressed cells was similar to that in wild type cells (2–4 foci, Figure 3D and 3E). Thus, we conclude that ManA is crucial for proper DNA organization and segregation during rapid growth.

### ManA is required for proper cell wall synthesis

**Figure 4:**
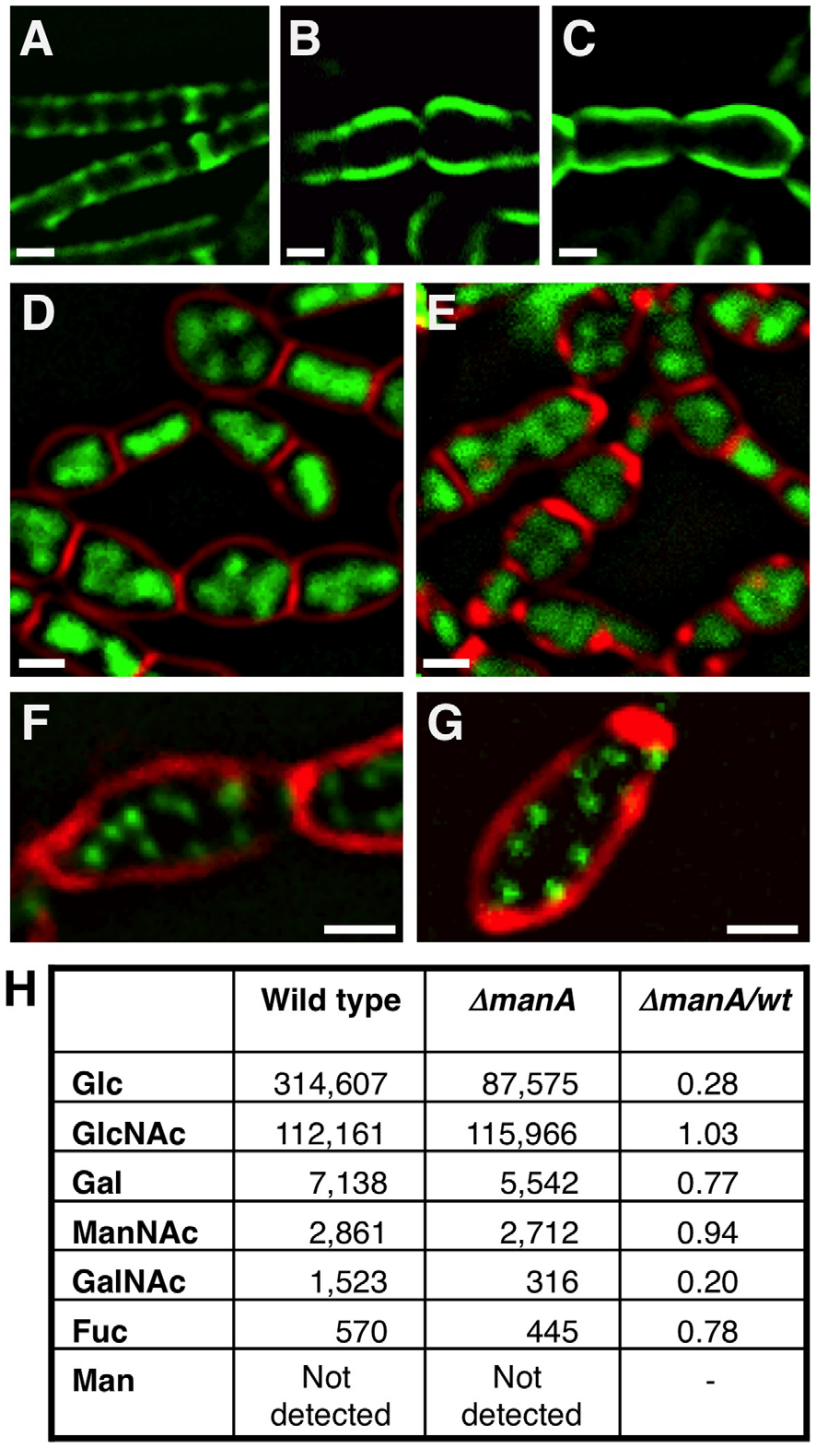
ManA participates in cell wall construction. (**A**–**C**) Fluorescence images of wild type (PY79) (A), Δ*manA* (ME37) (B), and tunicamycin (0.5 µg/ml) treated wild type (PY79) (C) cells labeled with WGA-FITC (green) (see Materials and Methods). (**D**–**E**) Fluorescence images of Δ*manA* (ME37) (D) and tunicamycin (0.5 µg/ml) treated wild type (PY79) (E) cells stained with DAPI (green) and FM1-43 (red). (**F**–**G**) Fluorescence images of Δ*manA* (ME34) (F) and tunicamycin (0.5 µg/ml) treated wild type (SB294) (G) cells producing Spo0J-GFP (green), stained with the membrane dye FM4-64 (red). (**H**) Cell walls of wild type (PY79) and Δ*manA* (ME37) cells were isolated, hydrolyzed and their glycosyl composition determined using HPAEC Neutral Monosaccharide Analysis (see Materials and Methods). The numbers (arbitrary units) represent the average obtained from three independent samples of each strain. For all panels, cells were grown in rich LB medium. Scale bars correspond to 1 µm.

As mentioned above, in association with abnormal nucleoids, Δ*manA* cells display morphological defects reminiscent of the phenotypes exhibited by cell wall mutants (e.g.: [6], [13], [15], [18]–[21], [29], [30]). This observation raised the idea that ManA is required for cell wall construction. To explore this possibility we visualized the cell wall architecture of Δ*manA* cells. Wild type and Δ*manA* cells were labeled with a fluorophore (fluorescein isothiocyanate - FITC) conjugated to wheat germ agglutinin (WGA), which is a carbohydrate binding protein that recognizes mainly GlcNAc. Living wild type cells stained with WGA-FITC exhibited bright midcell bands and fainter helical sidewall staining (Materials and Methods; Figure 4A). This non-uniform pattern is similar to the patterns reported when fixed *B. subtilis* cells are stained with WGA [9], [41] and when nascent PG is labeled with fluorescent vancomycin [5]. In contrast, living Δ*manA* cells stained with WGA-FITC displayed a much more homogeneous pattern (Figure 4B). The Δ*manA* cell wall appeared significantly thicker than the wild type cell wall and lacked the characteristic helical staining indicative of nascent PG. Examining the effect of ManA on the subcellular localization of the two cell wall components Mbl and TagO corroborated these findings. Mbl that is typically localized in a helical pattern [15] became dispersed, while the localization of the membrane-associated TagO [27] was hardly modified (Figure S4).

To substantiate that ManA is required for cell wall synthesis, we took advantage of the antibiotic tunicamycin to artificially interfere with cell wall construction and compare the resultant phenotypes with that of Δ*manA* cells. Tunicamycin is a uridine nucleoside analog that specifically binds to and blocks the first membrane-associated step of both PG and WTA biosynthesis and thus, actively inhibits cell wall synthesis [42], [43]. When wild type *B. subtilis* cells were grown in the presence of tunicamycin and subsequently visualized by fluorescence microscopy, a dramatic change in their cell shape was readily detected (Figure S5). Surprisingly, the tunicamycin treated cells resembled the Δ*manA* cells not only in their cell shape but also in their nucleoid morphologies (Figure 4D and 4E). Moreover, when wild type cells carrying the Spo0J-GFP fusion were treated with tunicamycin, a significant increase in the number of origins per cell was observed, indicating the transition to polyploidy (Figure 4F and 4G). Consistent with the finding that tunicamycin reproduces characteristic Δ*manA* phenotypes, wild type cells treated with tunicamycin and labeled with WGA-FITC displayed an architecture similar to the one exhibited by Δ*manA* cells (Figure 4C). Namely, the cell wall appeared thicker than usual and lacked typical sidewall cylindrical structures. Taken together, our data demonstrate that blocking cell wall synthesis per se by adding tunicamycin is sufficient to recapitulate both the cell shape and chromosome defects characteristic of Δ*manA* cells. Therefore, we surmise that ManA plays a significant role in cell wall construction.

### ManA influences the carbohydrate composition of the cell wall

Since ManA is primarily classified as a sugar metabolic enzyme, we reasoned that it could affect the carbohydrate composition of the cell wall. To test this possibility, cell walls of wild type and Δ*manA* cells were isolated, hydrolyzed and their glycosyl composition determined using HPAEC neutral monosaccharide analysis (Materials and Methods). The profile of wild type cell wall material was found to include high amounts of GlcNAc and Glc, medium levels of GalNAc and Gal, and relatively small quantities of Fucose (Fuc) (Figure 4H). These carbohydrates, though in different proportions, have been reported as cell wall constituents of pathogenic *Bacilli* species (i.e.: *B. cereus, B. anthracis,* and *B. thuringiensis*) [44], suggesting that these sugars are common to *bacilli*. The Δ*manA* cell wall contained the same carbohydrates, however, a significant decrease in the amounts of Glc (∼4 fold) and GalNAc (∼5 fold) was monitored, with a milder decrease in the levels of Gal and Fuc (6-deoxy-L-Galactose); these four carbohydrates are characteristic components of WTA [1]. In contrast, the major PG sugar GlcNAc was found to be present at the same level in wild type and Δ*manA* cells. Thus, ManA is required for proper formation of WTA and to a lesser degree for PG synthesis. Nevertheless, the modified architecture of the PG observed in the absence of ManA (Figure 4A and 4B) implies a tight coordination between WTA and PG construction.

### Linking cell wall integrity and chromosome morphology

**Figure 5:**
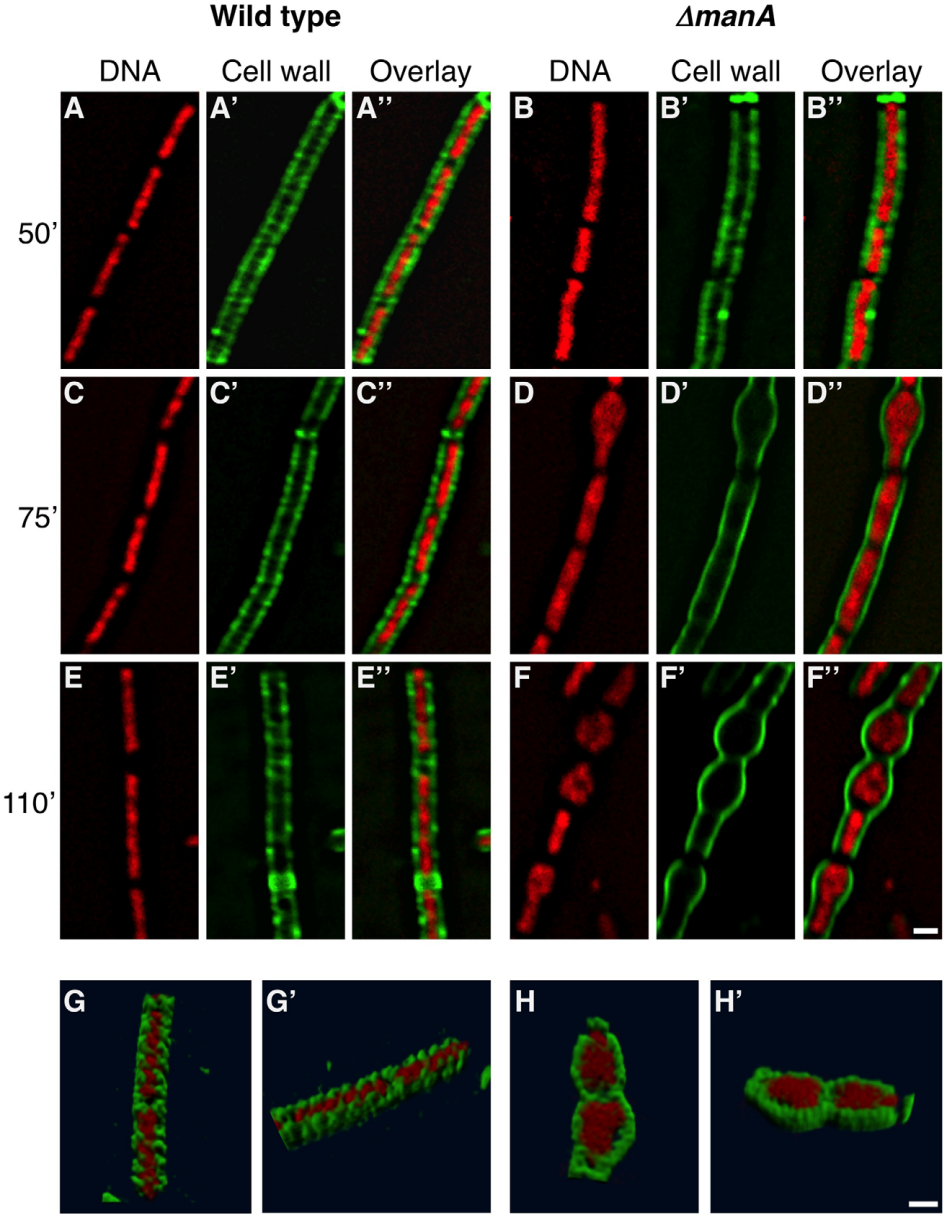
Following cell wall architecture and chromosome morphology. Wild type (PY79) and Δ*manA* (ME37) cells were grown in LB at 23°C and then shifted to 37°C. Samples were imaged at the indicated time points after temperature shift. (**A**–**F**) Superimposed fluorescence images of wild type (A, C, E) and Δ*manA* (B, D, F) cells labeled with WGA-FITC (green) and stained with DAPI (red). (**G**–**H**) 3D reconstruction (see Materials and Methods) of wild type (PY79) (G,G') and Δ*manA* (ME37) (H, H') cells labeled with WGA-FITC (green) and stained with DAPI (red) (see corresponding Videos S1 and S2, respectively). Scale bars correspond to 1 µm.

To better understand the connection between cell wall integrity and chromosome morphology we followed both components simultaneously. We took advantage of the observation that Δ*manA* cells exhibit almost normal phenotypes at a low temperature (23°C) but gradually evidence defects when shifted to a high temperature (37°C). Cell wall and nucleoid morphologies were followed by WGA-FITC labeling and DAPI staining, respectively, as the Δ*manA* cells were temperature shifted. In wild type cells the chromosome appeared mostly helical, exhibiting morphologies typical of replicating forms (Figure 5A, 5C and 5E) [45]. Notably, the helicity of the chromosome in the majority of the cells seemed to follow the sidewall staining of the cell wall. In a way, it seems that the cell wall restricts the nucleoid spatial localization by caging it. This feature was highlighted when stacks of optical sections were deconvolved, and three-dimensional structure was reconstructed (Figure 5G and Video S1). In comparison, the morphology of the nucleoids in Δ*manA* cells altered distinctively upon temperature shift. At the lower temperature the nucleoids appeared similar to those displayed by wild type cells (Figure 5B). However, after the temperature was shifted, concurrently with the cell bulging and loss of helical sidewall staining, the nucleoid lost its helical shape and became less compact and structured (Figure 5D and 5F). Moreover, as rods became spheres, the nucleoid expanded in a pattern associated with the new cell geometry, as if constraints confining its structure were being released (Figure 5H and Video S2). It is possible that the incapability to confine the chromosome leads to defects in DNA segregation, ultimately resulting in the formation of polyploid cells.

Consistent with a putative relationship between cell wall integrity and chromosome morphology, polyploidy was observed in L-form mutant cells, which completely lack a cell wall [46], [47]. Additionally, an examination of chromosome copy number in the cell wall mutant *mreB* in *E. coli* revealed multiple chromosomal copies per cell [21]. In accord, *B. subtilis* cells harboring mutations in *mreB* or *mbl*, or wild type cells treated with tunicamycin showed multiple chromosomal copies (Figure 4F and 4G; Figure S6). Thus, an intimate connection between cell wall integrity and chromosome morphology exists. Perturbing this connection gives rise to the formation of polyploid cells.

## Discussion

Cell wall is responsible for shape determination and cellular viability for most bacterial species. Here we demonstrate that the carbohydrate metabolic enzyme ManA, which is conserved among prokaryotes and eukaryotes, participates in cell wall construction of the Gram positive bacterium *B. subtilis*. Several lines of evidence demonstrate a direct connection between ManA and cell wall synthesis: 1) the shape defect and perturbed cell wall architecture exhibited by Δ*manA* cells, 2) the resemblance between the phenotype of Δ*manA* cells and that of wild type cells treated with the cell wall synthesis inhibitor tunicamycin and, 3) the decreased amount of specific carbohydrates coating the cell surface observed in the absence of ManA.

What could be the role of ManA in cell wall synthesis? ManA is a cytoplasmic enzyme that catalyzes the reversible isomerization of Fru-6-P and Man-6-P [32]. Both products could affect directly cell wall synthesis. Man-6-P is a substrate for generating GDP-mannose, which is an important precursor of many nucleotide sugars, such as GDP-fucose [48], whereas Fru-6-P is a component of a pathway that leads to the formation of the nucleotide sugar UDP-GlcNAc, basic for PG assembly [2]. Thus, the absence of ManA enzyme could interfere with the equilibrium of several pathways producing nucleotide sugar reservoirs for PG and WTA synthesis. Consistently, deleting the *pgi* gene encoding an enzyme that produces Fru-6-P [49] resulted in phenotypes similar to Δ*manA* (Figure S7). This notwithstanding, according to our observations the absence of ManA has a specific effect on the composition of cell wall carbohydrates: it causes reduced abundance of Glc and GalNAc without perturbing the levels of the major PG precursor GlcNAc. Since, both Glc and GalNAc are components of the teichoic acid pathway [1], our data suggest that ManA is specifically involved in the WTA synthesis rather than in the PG pathway. Moreover, although the overall composition of the PG components was unaffected, visualization of the PG revealed a modified architecture implying that the balance between PG and WTA is crucial for proper cell wall construction.

Interestingly, in some cases mannose phosphate isomerases have been reported to affect cellular structures in other microorganisms. For example, the ManA enzyme of the bacterium *Helicobacter pylori* participates in capsular biosynthesis [48], while the eukaryotic fungus *Aspergillus fumigatus* displays altered cell wall synthesis and morphogenesis under mannose starvation conditions [50].

**Figure 6:**
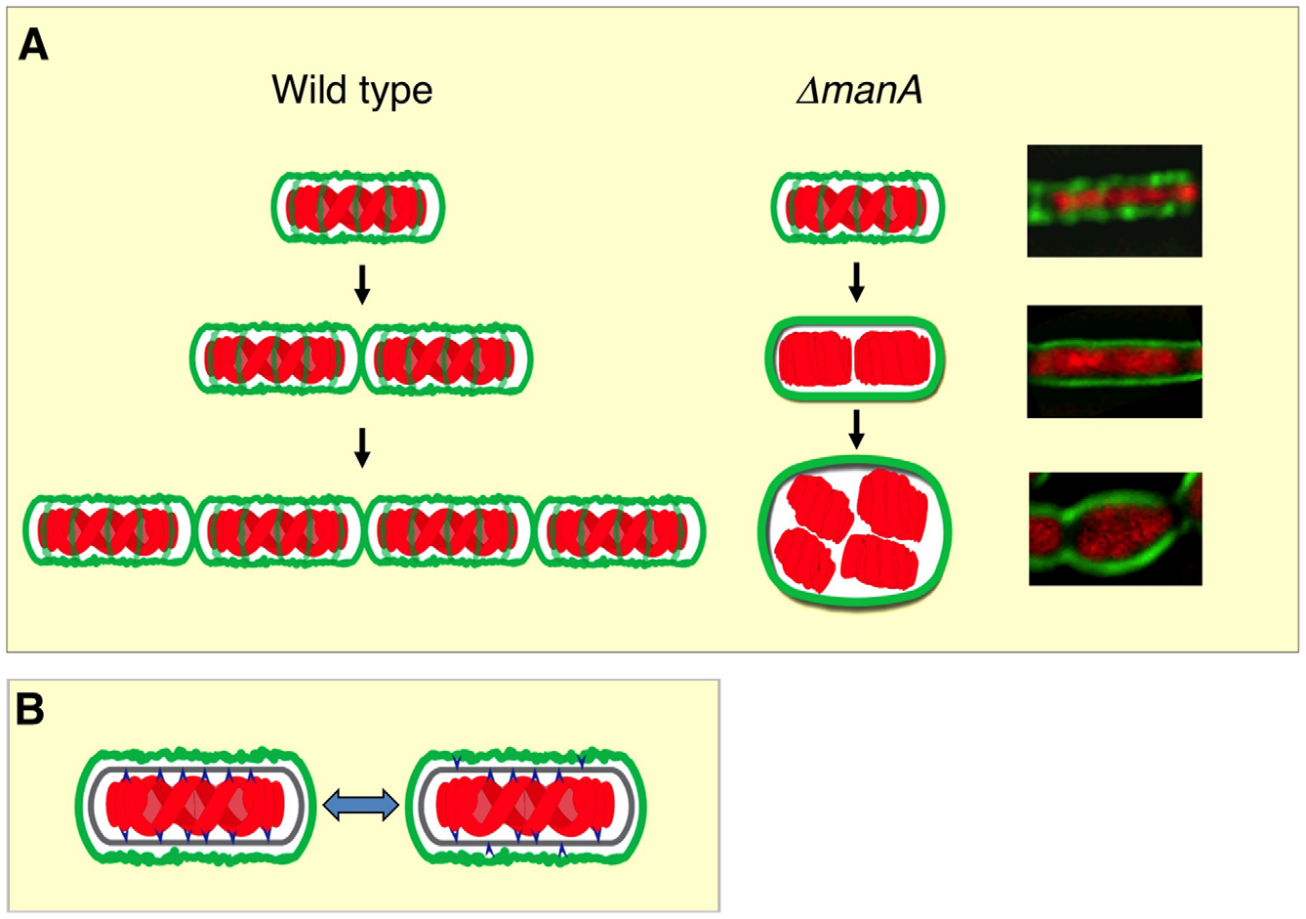
Linking cell wall integrity and chromosome morphology. (**A**) A model illustrating the interaction between cell wall (green) and chromosome (red) in wild type and Δ*manA* cells. Three generations of wild type (left panels) and Δ*manA* (middle panels) cells are depicted. Corresponding fluorescence images of Δ*manA* cells (right panels) are shown. (**B**) The cell wall lipid intermediate flip-flop reaction is an active mechanism for coordinating chromosome organization with cell wall elongation. Shown are chromosome (red), cell wall (green), cell wall lipid intermediates (blue) and membrane (gray). Chromosome and cell wall lipid intermediates undergo attaching/detaching cycles depending on the side of the membrane the lipid intermediates are present.

We have previously shown that the bacterial nucleoid adopts a helical morphology during DNA replication [45]. Here we demonstrate that this nucleoid architecture is strictly dependent on cell wall integrity (Figure 5; Video S1 and S2). Interestingly, it has been proposed that the bacterial chromosome structure is largely affected by the transcription of rRNA operons and the transcription-translation-insertion (transertion) of membrane proteins that fasten the chromosome to the membrane [51], [52]. Thus, it is conceivable that the chromosome is anchored to, and coordinated with both components cell wall and membrane. The association between DNA morphology and the cell wall is strongly manifested by the high chromosome copy number observed in L-form [46], [47], tunicamycin treated cells (this work), *mreB* and *mbl* mutants [21] (this work). We propose a model whereby the insertion of new cell wall material in a helical pattern dictates chromosome helicity (Figure 6A). In wild type cells the nucleoid is attached to the newly synthesized cell wall (see below) and its organization and segregation are coordinated with cell wall synthesis and elongation. In the absence of ManA the normal extension of the cell wall is blocked, as indicated by the disappearance of helical sidewall staining. Consequently, the nucleoid is detached from cell wall components and the synchronization is lost between cell growth and DNA replication and segregation, resulting in the formation of polyploid cells. When detached, the nucleoid loses its compact helical structure and adopts a looser conformation that seems to follow the overall cellular geometry.

It still remains to be resolved how the nucleoid is attached to the newly synthesized cell wall. Cell wall synthesis initiates on the cytoplasmic side of the membrane and then cell wall precursors are translocated to the outer membrane side [2]. This flip-flop reaction could be an active mechanism for coordinating DNA organization with cell wall elongation. We propose that the DNA is attached to the newly synthesized lipid intermediates on the cytoplasmic side of the membrane. When lipid II flips across the membrane the bound DNA is released, and the detached DNA is now free to bind a new cytoplasmic cell wall precursor (Figure 6B). These attaching/detaching cycles serve to coordinate DNA replication, segregation and cell wall formation. When cell wall construction is blocked the amount of new cytoplasmic cell wall precursors is reduced. Therefore the detached chromosome falls off the membrane and the linkage between cell wall and DNA is lost.

The interaction between the cell wall machinery and the DNA could be also mediated by linker membrane proteins that bind to both DNA and the cell wall. An interesting candidate proposed to possess such a capability is RodZ [53], a bacterial cell morphogenesis protein identified recently in *E. coli* [54], [55], *C. crescentus* and *B. subtilis* [56]. RodZ contains a transmembrane domain and a cytosolic helix-turn-helix (HTH) DNA-binding motif and was shown to form helical structures and associate with MreB [54]–[56]. An additional membrane protein reported to colocalize with MreB and affect nucleoid morphology is SetB in *E. coli* [57]. Importantly, the primary role of SetB is metabolic, as it was shown to act as a lactose and glucose efflux transporter [58]. It is possible that metabolic proteins such as ManA and SetB through their role in cell wall synthesis operate as sensors that synchronize cell wall elongation with metabolite availability. Indeed, a similar ‘coupling’ function between cell mass and cell division has been attributed to the metabolic protein UgtP, a sugar transferase in *B. subtilis* that acts both in WTA biosynthesis and inhibits assembly of the cell division protein FtsZ [59].

The similarity between cell wall biosynthesis in bacteria and protein N-linked glycosylation in eukaryotes, a process which is utilized to determine the rate of protein folding [60], [61], raises the possibility that cell wall elongation serves to time cellular activities. In eukaryotes, N-linked glycan chains are added in the endoplasmic reticulum to growing nascent polypeptides and promote proper protein folding [62]. In both N-linked glycosylation and cell wall biosynthesis the initial step is transfer of a sugar nucleotide to a lipid carrier, undecaprenyl phosphate in bacteria and dolichol phosphate in eukaryotes [61], reactions that are inhibited by the antibiotic tunicamycin [42], [63]. During N-linked glycosylation the sugar chain, containing mainly mannose, is transferred from the lipid carrier to the unfolded protein. This sugar tree then undergoes cycles of cleavage and synthesis that serve as a timer for reporting the state of protein folding [64]. In a similar manner, we propose that the cell wall elongation rate in bacteria serves as a timer that coordinates cell growth with critical cellular activities such as chromosome segregation and cell division.

## Materials and Methods

### Strains and general methods

Plasmid and primers used for this study are described in Text S1 and Table S1. *B. subtilis* strains were derivatives of the wild type strain PY79 [65] and are listed in Table S2. All general methods were carried out as described previously [66]. Cultures were inoculated at 0.05 OD_600_ from an overnight culture and growth was carried out at 23°C, 37°C or 42°C, in LB medium or in S7 minimal medium [67] as indicated.

### Transposon mutagenesis

Mini-Tn*10* transposon was inserted into the *B. subtilis* (PY79) chromosome as described previously [68], [69]. Out of 2700 colonies that were screened, 260 that showed atypical colony morphologies (i.e.: smooth colonies, transparent colonies) and/or growth defect (i.e.: small colonies) were selected for further analysis.

### Prediction of ManA active site

Using UniProtKB/Swiss-Prot (http://www.uniprot.org/uniprot/O31646), the active site of ManA was determined largely by comparison with the crystal-solved ortholog Pmi from *Candida albicans.* Accordingly, two amino acids were chosen to be examined: histidine 97 which is located within a Zinc ion binding site, and the highly conserved arginine 192, which is predicted to be part of the catalytic domain.

### Fluorescence microscopy

Fluorescence microscopy was carried out as described previously [70]. Briefly, samples (0.5 ml) were taken during logarithmic phase, centrifuged and resuspended in 10 µl of PBS×1 supplemented with the fluorescent membrane stain FM1-43 or FM4-64 (Molecular Probes, Invitrogen) at 1 µg/ml and the DNA stain 4,6-Diamidino-2-phenylindole (DAPI) (Sigma) at 2 µg/ml. For cell wall labeling, cells were harvested, gently centrifuged, resuspended in 100 µl of T-Base×1 supplemented with WGA-FITC (5 µg/ml, Sigma), incubated for 15 min at room temperature, and washed twice with T-Base×1 before imaging. Stained cell walls and GFP fused proteins were visualized by placing the cells on thin T-Base×1 1% agarose pads. Cells were visualized and photographed using an Axioplan2 microscope (Zeiss) equipped with a CoolSnap HQ camera (Photometrics, Roper Scientific) or an Axioobserver Z1 microscope (Zeiss) equipped with a CoolSnap HQII camera (Photometrics, Roper Scientific). System control and image processing were performed using MetaMorph software (Molecular Devices). For deconvolution microscopy, samples of cells grown in rich LB medium were labeled with WGA-FITC (30 µg/ml) and DAPI (2 µg/ml), applied to an agarose pad, and then subjected to deconvolution microscopy. Optical sections (20–40) were collected at a spacing of 0.2 µm. Images were deconvolved through 50 iterations and then visualized as SFP volume render by using the Huygens Professional Software (Scientific Volume Imaging b.v., Netherlands).

### Determining cell wall carbohydrate composition

Cell walls of wild type (PY79) and Δ*manA* (ME37) cells (triplicates of 250 ml late logarithmic phase cultures) were isolated as described previously [44]. The analysis was performed by GlycoSolutions Corporation (MA, USA). Briefly, each of the lyophilized samples was resuspended in 250 µl sterile double distilled water, rinsed with 250 µl of 4 M trifluoroacetic acid. Of note, by using this method acidic carbohydrates such as MurNAc cannot be indentified. After 3 hours of hydrolysis samples were examined using GlycoSolutions SOP HPAEC Neutral Monosaccharide Analysis. Equal volumes of each sample were injected at various dilutions: undiluted, x10, x100. ManNAc and glucose standard curves were included to normalize the values.

### Microarray analysis

DNA Microarray analysis was preformed to determine the DNA content of wild type (PY79) and Δ*manA* (ME37). Digested genomic DNA (0.5 µg, *Hae*III) of each strain was amplified by random primer and then labeled indirectly with cy3 or cy5 dyes. Equal amounts of the labeled samples were mixed and hybridized to a DNA chip containing Sigma *B. subtilis* OligoLibrary, which represents the entire *B. subtilis* ORFs. Arrays were scanned using a Genepix 4000B scanner (Axon Ltd). Fluorescence intensities were quantitatively analyzed using GenePix Pro 4.1 software (Axon).

## Supporting Information

Figure S1 **Complementation of Δ** ***manA*** **phenotype by ectopic expression.** Fluorescence microscopy was carried out on: (**A**) wild type cells (PY79), (**B**) Δ*manA* cells (ME37), (**C**) Δ*manA* cells harboring *P_manA_-manA* (ME42), (**D**) Δ*manA* cells harboring *P_manA_-manA_H97A_* (ME162), and (**E**) Δ*manA* cells harboring *P_manA_-manA_R192A_*. (ME163). Cells were grown in rich LB medium. DNA and membrane were visualized with DAPI (green) and FM1-43 (red), respectively. Scale bar corresponds to 1 µm.
(2.49 MB TIF)

Figure S2 **Expression of ManA-GFP and Pmi-GFP during growth in rich LB medium.** Fluorescence microscopy was carried out on wild type (PY79), *manA-gfp* (ME48) and *pmi-gfp* (ME134) cells grown in rich LB medium. (**A**) Background fluorescence of wild type cells lacking *gfp*. (**B**) Visualization of *manA-gfp* (green). (**C**) *pmi-gfp* was undetectable. Scale bar corresponds to 1 µm.
(0.45 MB TIF)

Figure S3 **Δ** ***manA*** **phenotype is growth rate dependent.** **I.** Fluorescence microscopy was carried out on Δ*manA* (ME37) cells grown in rich LB medium at different temperatures. DNA and membrane were visualized with DAPI (green) and FM1-43 (red), respectively. (**A**) Δ*manA* cells grown at 37°C. (**B**) Δ*manA* cells grown at 23°C. **II.** Growth curves of wild type (PY79) and Δ*manA* (ME37) strains grown in minimal S7 medium, or when shifted from minimal medium to rich LB medium, as indicated. **III.** Time course microscopy of cells producing ManA-GFP (ME48) (green) shifted from minimal medium (t = 0) to rich LB medium. (**A**) t = 0 minutes, (**B**) t = 60 minutes, and (**C**) t = 120 minutes. Scale bars correspond to 1 µm.
(2.49 MB TIF)

Figure S4 **Investigating the effect of ManA on localization of cell wall proteins.** TagO-GFP and GFP-Mbl were visualized in wild type or Δ*manA* cells grown in rich LB medium. For *P_xyl_-gfp-mbl* induction 1% xylose was added. (**A**) ME141 cells (*tagO-gfp*) (**B**) ME145 cells (*tagO-gfp,* Δ*manA*) (**C**) ME143 cells (*P_xyl_-gfp-mbl*) (**D**) ME147 cells (*P_xyl_-gfp-mbl,* Δ*manA*). Signal from TagO-GFP or GFP-Mbl is shown in green. Scale bar corresponds to 1 µm.
(1.19 MB TIF)

Figure S5 **Time course analysis of tunicamycin treated cells.** Time course microscopy was carried out on wild type (PY79) cells grown in rich LB medium supplemented with 0.5 µg/ml tunicamycin. Tunicamycin was added to the culture at OD_600_ = 0.2 (t = 0). (**A**) t = 0 minutes, (**B**) t = 30 minutes, (**C**) t = 60 minutes, and (**D**) t = 90 minutes. DNA and membrane were visualized with DAPI (green) and FM1-43 (red), respectively. Scale bar corresponds to 1 µm.
(1.74 MB TIF)

Figure S6 **Polyploidy is found in different cell wall mutants.** Origin region was visualized in *mbl* and *mreB* mutant cells producing Spo0J-GFP grown in rich LB medium. (**A**) ME89 cells (*mbl::mls, spoOJ-gfp-spc-cat)* **(B)** ME136 cells [*trpC2Ω(amyE::Pxyl-c-myc-mreBCD-spc)Ω(mreB::neo), spo0J-gfp-spc-cat*] depleted for MreB (for MreB depletion: cells were grown on 1% xylose containing plate, and then transferred to liquid LB medium without xylose). Spo0J-GFP signal is shown in green, and membrane dye FM4-64 is shown in red. Scale bar corresponds to 1 µm.
(0.49 MB TIF)

Figure S7 **Δ** ***pgi*** **cells exhibit phenotypes similar to** Δ***manA*** **cells.** Cells were grown in rich LB medium and observed by fluorescence microscopy. (**A**) ME138 (Δ*pgi*) cells stained with DAPI (green) and FM1-43 (red). (**B**) ME139 (Δ*pgi*) cells producing Spo0J-GFP (green) were stained with FM4-64 (red). Scale bar corresponds to 1 µm.
(0.77 MB TIF)

Table S1 **List of primers.**
(0.05 MB DOC)

Table S2 ***B. subtilis*** **strains used in this study.**
(0.08 MB DOC)

Text S1 **Supporting materials and methods.**
(0.04 MB DOC)

Video S1 **3D reconstruction of wild type (PY79) cells labeled with WGA-FITC (green) and stained with DAPI (red) (see** **Materials and Methods** **).**
(1.40 MB AVI)

Video S2 **3D reconstruction of Δ** ***manA*** **(ME37) cells labeled with WGA-FITC (green) and stained with DAPI (red) (see** **Materials and Methods** **).**
(1.15 MB AVI)
