# Supplementary figures and images for: The Metabolic Enzyme ManA Reveals a Link between Cell Wall Integrity and Chromosome Morphology
Source: PLoS Genet. 2010 Sep 16;6(9):e1001119. doi: 10.1371/journal.pgen.1001119 (PMC2940726; doi:10.1371/journal.pgen.1001119)

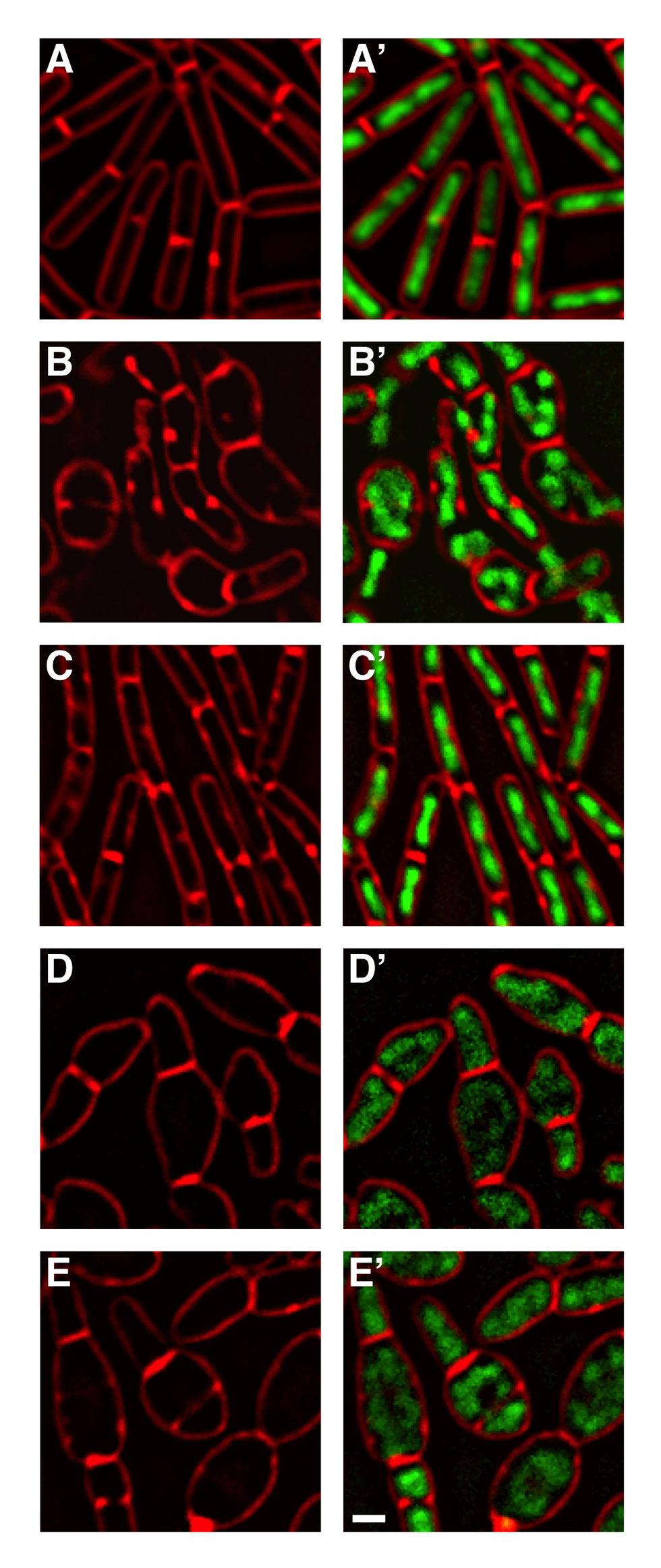

Supplement: Figure S1 — Complementation of Δ manA phenotype by ectopic expression. Fluorescence microscopy was carried out on: (A) wild type cells (PY79), (B) ΔmanA cells (ME37), (C) ΔmanA cells harboring PmanA-manA (ME42), (D) ΔmanA cells harboring PmanA-manAH97A (ME162), and (E) ΔmanA cells harboring PmanA-manAR192A. (ME163). Cells were grown in rich LB medium. DNA and membrane were visualized with DAPI (green) and FM1-43 (red), respectively. Scale bar corresponds to 1 µm. (2.49 MB TIF) [file pgen.1001119.s001.tif]

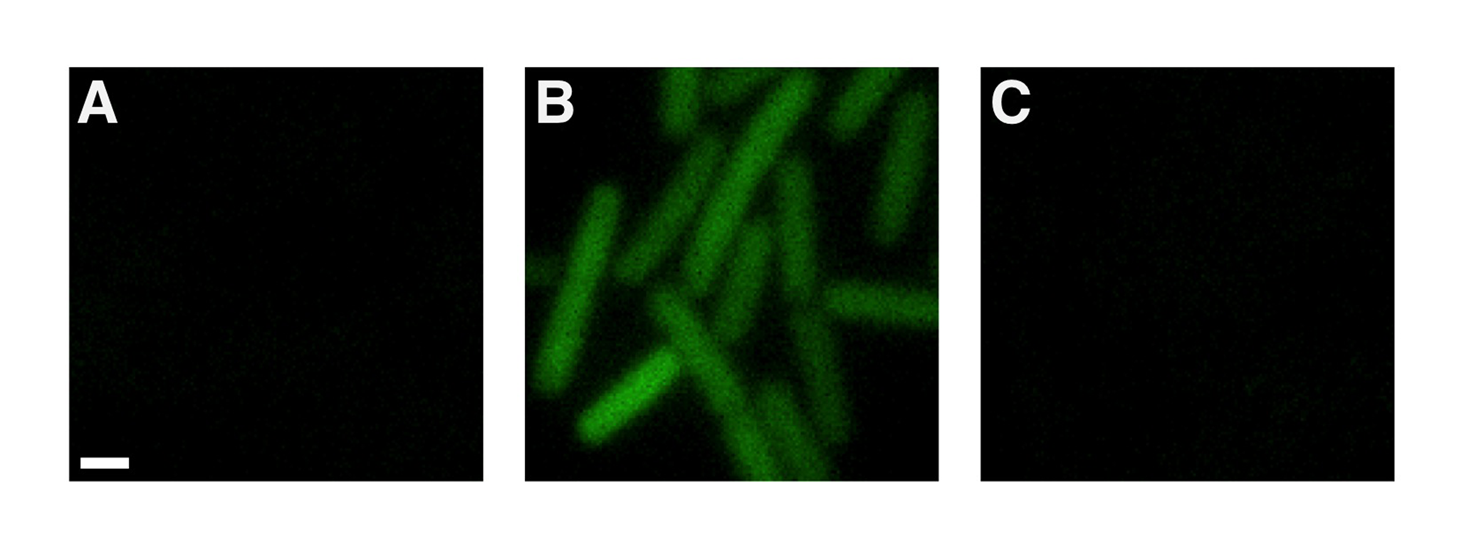

Supplement: Figure S2 — Expression of ManA-GFP and Pmi-GFP during growth in rich LB medium. Fluorescence microscopy was carried out on wild type (PY79), manA-gfp (ME48) and pmi-gfp (ME134) cells grown in rich LB medium. (A) Background fluorescence of wild type cells lacking gfp. (B) Visualization of manA-gfp (green). (C) pmi-gfp was undetectable. Scale bar corresponds to 1 µm. (0.45 MB TIF) [file pgen.1001119.s002.tif]

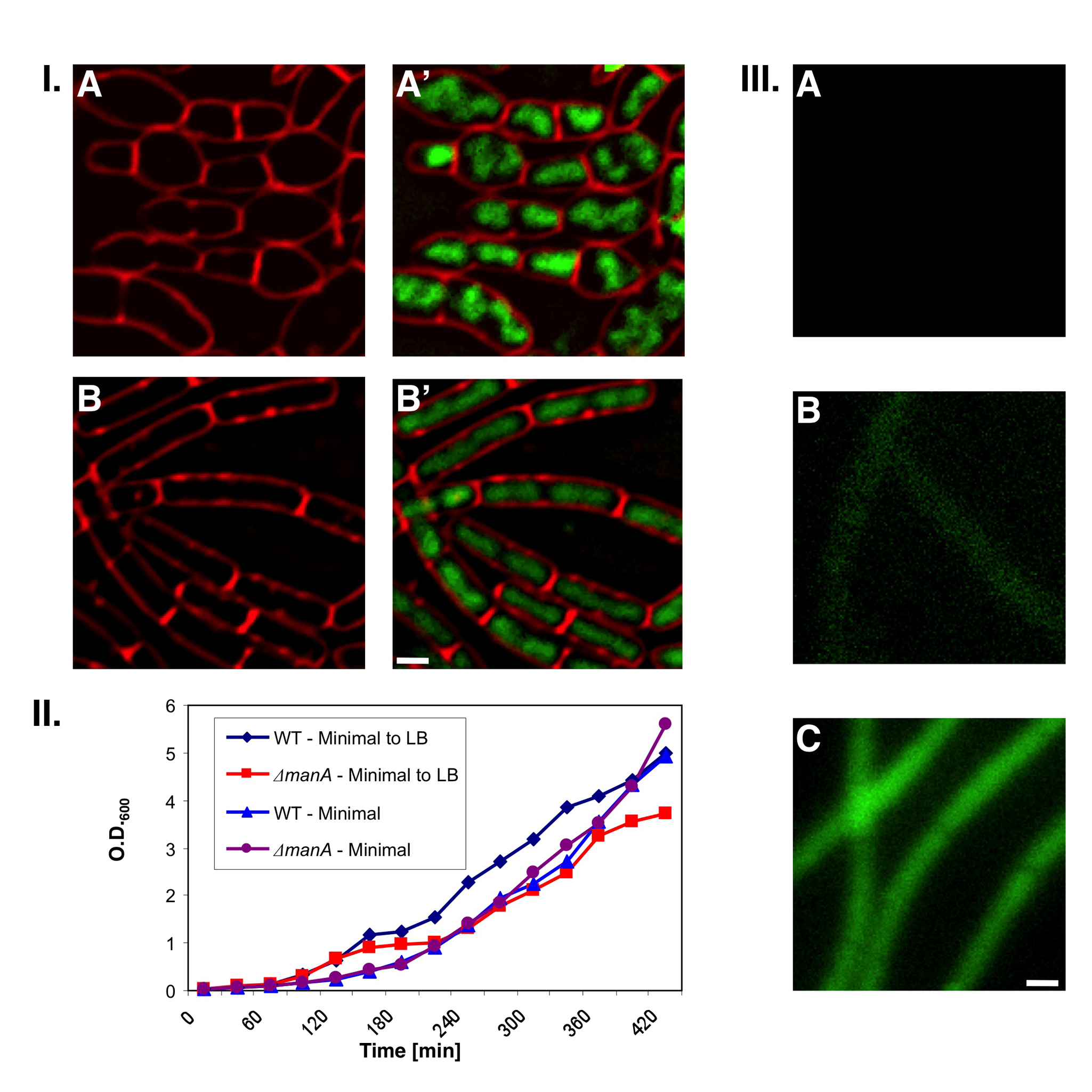

Supplement: Figure S3 — Δ manA phenotype is growth rate dependent. I. Fluorescence microscopy was carried out on ΔmanA (ME37) cells grown in rich LB medium at different temperatures. DNA and membrane were visualized with DAPI (green) and FM1-43 (red), respectively. (A) ΔmanA cells grown at 37°C. (B) ΔmanA cells grown at 23°C. II. Growth curves of wild type (PY79) and ΔmanA (ME37) strains grown in minimal S7 medium, or when shifted from minimal medium to rich LB medium, as indicated. III. Time course microscopy of cells producing ManA-GFP (ME48) (green) shifted from minimal medium (t = 0) to rich LB medium. (A) t = 0 minutes, (B) t = 60 minutes, and (C) t = 120 minutes. Scale bars correspond to 1 µm. (2.49 MB TIF) [file pgen.1001119.s003.tif]

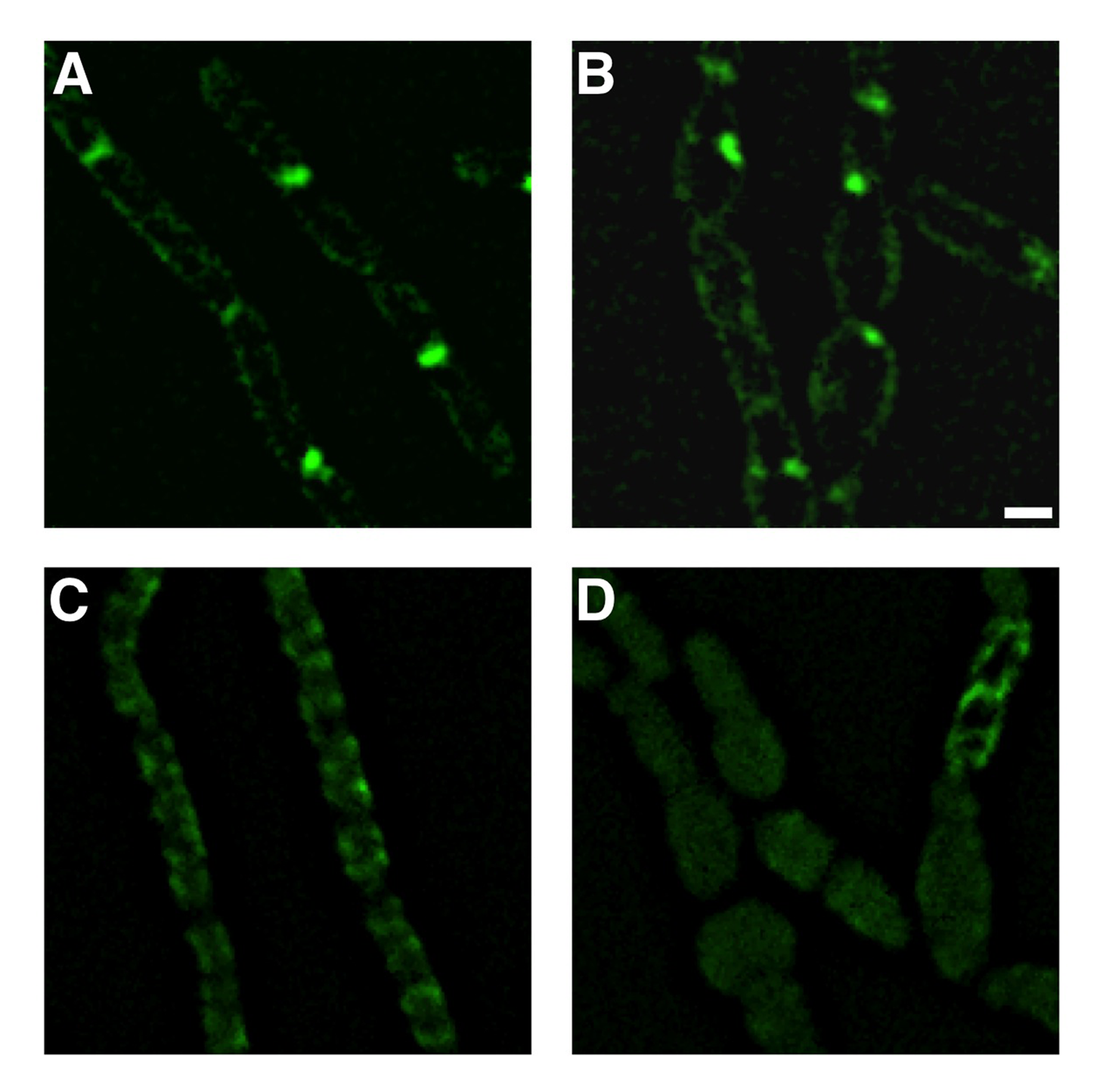

Supplement: Figure S4 — Investigating the effect of ManA on localization of cell wall proteins. TagO-GFP and GFP-Mbl were visualized in wild type or ΔmanA cells grown in rich LB medium. For Pxyl-gfp-mbl induction 1% xylose was added. (A) ME141 cells (tagO-gfp) (B) ME145 cells (tagO-gfp, ΔmanA) (C) ME143 cells (Pxyl-gfp-mbl) (D) ME147 cells (Pxyl-gfp-mbl, ΔmanA). Signal from TagO-GFP or GFP-Mbl is shown in green. Scale bar corresponds to 1 µm. (1.19 MB TIF) [file pgen.1001119.s004.tif]

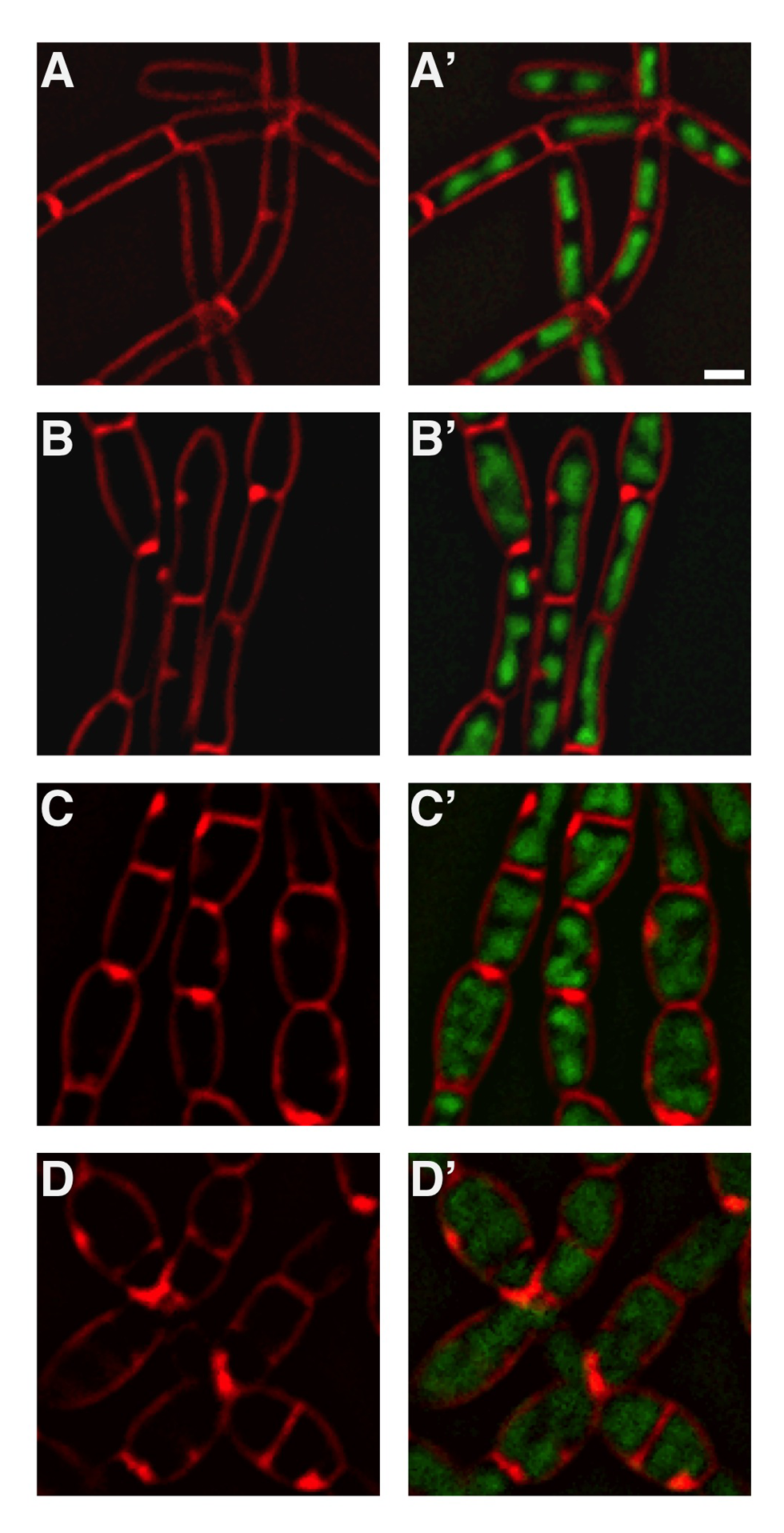

Supplement: Figure S5 — Time course analysis of tunicamycin treated cells. Time course microscopy was carried out on wild type (PY79) cells grown in rich LB medium supplemented with 0.5 µg/ml tunicamycin. Tunicamycin was added to the culture at OD600 = 0.2 (t = 0). (A) t = 0 minutes, (B) t = 30 minutes, (C) t = 60 minutes, and (D) t = 90 minutes. DNA and membrane were visualized with DAPI (green) and FM1-43 (red), respectively. Scale bar corresponds to 1 µm. (1.74 MB TIF) [file pgen.1001119.s005.tif]

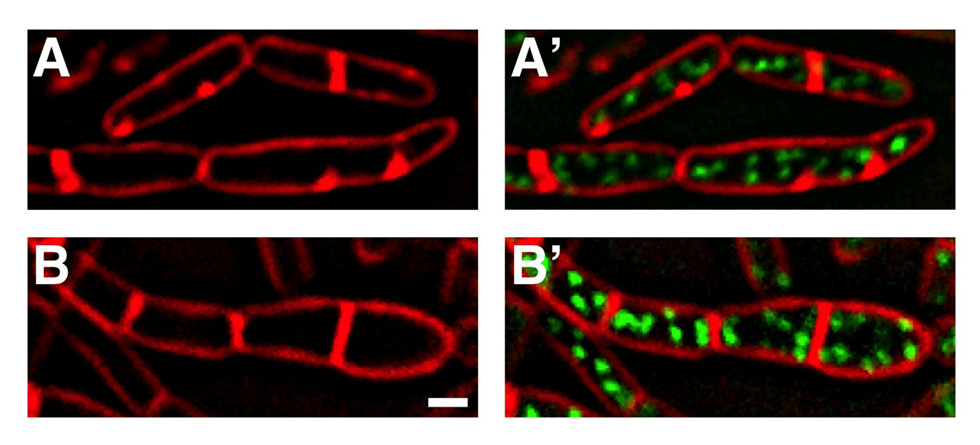

Supplement: Figure S6 — Polyploidy is found in different cell wall mutants. Origin region was visualized in mbl and mreB mutant cells producing Spo0J-GFP grown in rich LB medium. (A) ME89 cells (mbl::mls, spoOJ-gfp-spc-cat) (B) ME136 cells [trpC2Ω(amyE::Pxyl-c-myc-mreBCD-spc)Ω(mreB::neo), spo0J-gfp-spc-cat] depleted for MreB (for MreB depletion: cells were grown on 1% xylose containing plate, and then transferred to liquid LB medium without xylose). Spo0J-GFP signal is shown in green, and membrane dye FM4-64 is shown in red. Scale bar corresponds to 1 µm. (0.49 MB TIF) [file pgen.1001119.s006.tif]

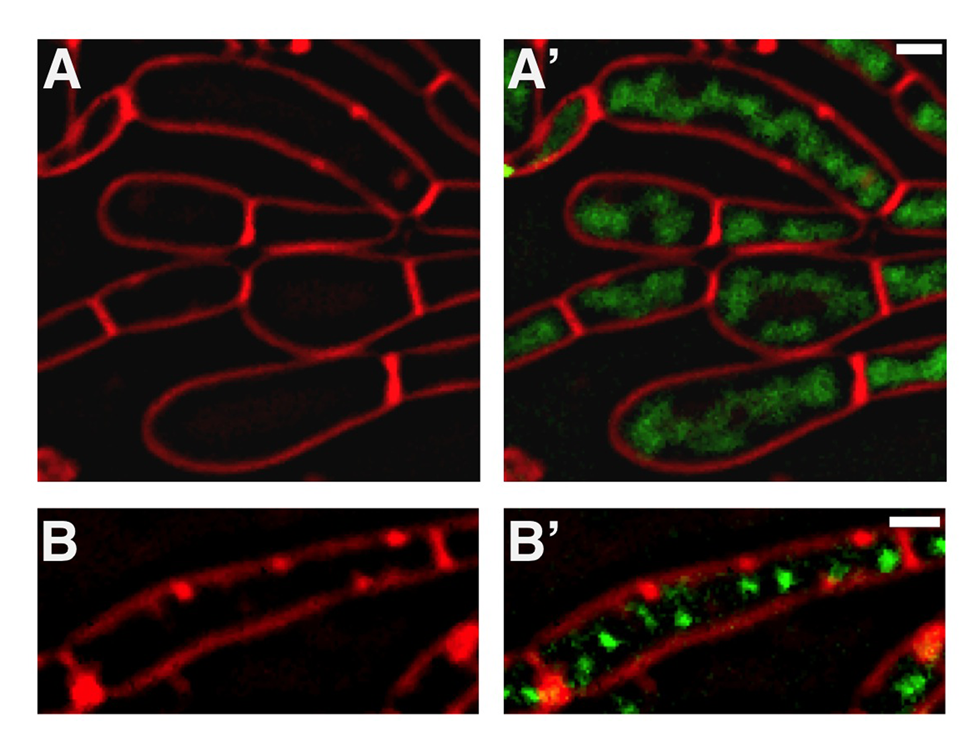

Supplement: Figure S7 — Δ pgi cells exhibit phenotypes similar to ΔmanA cells. Cells were grown in rich LB medium and observed by fluorescence microscopy. (A) ME138 (Δpgi) cells stained with DAPI (green) and FM1-43 (red). (B) ME139 (Δpgi) cells producing Spo0J-GFP (green) were stained with FM4-64 (red). Scale bar corresponds to 1 µm. (0.77 MB TIF) [file pgen.1001119.s007.tif]
