# Supplementary material for: The Metabolic Enzyme ManA Reveals a Link between Cell Wall Integrity and Chromosome Morphology
Source: PLoS Genet. 2010 Sep 16;6(9):e1001119. doi: 10.1371/journal.pgen.1001119 (PMC2940726; doi:10.1371/journal.pgen.1001119)
Supplement: Table S1 — List of primers. (0.05 MB DOC) [file pgen.1001119.s008.doc]

**Table S1. List of primers**

| **Primer name** | **Primer sequence** |
| --- | --- |
| **198** | 5'-cgtgataaatggtgggttcactcc-3' |
| **232** | 5'-tggatcggcaatgattggagaagg-3' |
| **233** | 5'-atcacctcaaatggttcgctgggtttccccgctttattcgatttcttctag-3' |
| **234** | 5'-aagttcgctagataggggtcccgagcccatctgtaacatggcagggcttg-3' |
| **255** | 5'-gcctaggatccgcagggattattccttgctttttttg-3' |
| **256** | 5'-ccggaattcccggcggccgcaggaaacctcctttaaagt gtg-3' |
| **257** | 5'-ccggcggccgcatgacgactgaaccgttatttttcaag-3' |
| **258** | 5'-cccgcaattgcaaattta tttctcaagccctgcc-3' |
| **277** | 5'-cccgcaattgtagagcgtggagaatgggatg-3' |
| **278** | 5'-aagtccgctcgagcaga tgggagacgatacattc-3' |
| **790** | 5'-aagtccgctcgagaatatgagacacgataagggtacaag-3' |
| **791** | 5'-acctaggaattctgctggtacattatcgactgtaag-3' |
| **975** | 5'-cggaggcagtgtgattgactg-3' |
| **976** | 5'-attatgtcttttgcgcagtcggcgctgctgtccctccataacgg-3' |
| **977** | 5'-cattcaattttgagggttgccagtaatgtgagaaagctgactggc-3' |
| **978** | 5'-ttcccctcctcttacatacaag-3' |
| **985** | 5'-acctaggaattcacctgattgatggattggatggcc-3' |
| **986** | 5'-aagtccgctcgagattccttttcaccagccgttt-3' |
| **987** | 5'-acctactgcggccgcatgtttgcaagggatattggtatt-3' |
| **988** | 5'-gcctaggatcccgtcatttaacatcttttcgtgaaggc-3' |
| **993** | 5'-acttatctgttcaggtggcgccgaatgatgaatatgc-3' |
| **994** | 5'-gcatattcatcattcggcgccacctgaacagataagt-3' |
| **995** | 5'-actcagacacaacctacgcattatatgattatgaccg-3' |
| **996** | 5'-cggtcataatcatataatgcgtaggttgtgtctgagt-3' |
