# Supplementary material for: The Metabolic Enzyme ManA Reveals a Link between Cell Wall Integrity and Chromosome Morphology
Source: PLoS Genet. 2010 Sep 16;6(9):e1001119. doi: 10.1371/journal.pgen.1001119 (PMC2940726; doi:10.1371/journal.pgen.1001119)
Supplement: Table S2 — B. subtilis strains used in this study. (0.08 MB DOC) [file pgen.1001119.s009.doc]

**Table S2. *B. subtilis* strains used in this study**

| **Strain** | **Genotype** | **Comments** |
| --- | --- | --- |
| PY79 | Wild type | [1] |
| SB294 | *spo0J-gfp-spc-cat* | A gift from Alan Grossman [2] |
| ME20 | *manA::Tn10-spc* |  |
| ME34 | *spoOJ-gfp-spc-cat, manA::Tn10-spc* |  |
| ME37 | *manA::kan* | The ORF of *manA* (codons 1-938) was replaced by *kan* gene using a long-flanking-homology PCR [3]*. |
| ME42 | *amyE::P_manA_-manA-cat, manA::kan* | *amyE::P_manA_-manA-cat* allele was constructed using plasmid pME15. |
| ME46 | *dnaB134*(ts) *zhb-83::Tn917-erm, manA::kan* | *dnaB134*(ts) allele, a gift from Alan Grossman. |
| ME48 | *manA-gfp-spc* | *manA-gfp-spc* allele was constructed using plasmid pME17. |
| ME79 | *veg::tetR-gfp-erm*,  *130(ykpA-B)ΩpKM218a(tetO)120(cat)* | [4] |
| ME82 | *veg::tetR-gfp-erm*,  *130(ykpA-B)ΩpKM218a(tetO)120(cat), manA::kan* |  |
| ME89 | *mbl::erm, spoOJ-gfp-spc-cat* | [5] |
| ME133 | *dnaB134*(ts) *zhb-83::Tn917-erm*,  *spo0J-gfp-spc-cat*, *manA::kan* |  |
| ME134 | *pmi-gfp-spc* | *pmi-gfp-spc* allele was constructed using plasmid pME25 |
| ME136 | *trpC2Ω(amyE::Pxyl-c-myc-mreBCD-spc)Ω(mreB::neo), spo0J-gfp-spc-cat* | *mreB* mutant, a gift from J. Errington. |
| ME138 | *pgi::erm* | The ORF of *pgi* (codons 1-1353) was replaced by *erm* gene using a long-flanking-homology PCR [3]**. |
| ME139 | *pgi::erm,* *spoOJ-gfp-spc-cat* |  |
| ME141 | *tagO-gfp-spc* | *tagO -gfp-spc* allele was constructed using plasmid pME28. |
| ME143 | *amyE::P_xyl_-gfp-mbl-cat* | *AmyE:: P_xyl_-gfp-mbl-cat* allele was constructed using plasmid pME29. |
| ME145 | *tagO-gfp-spc, manA::kan* |  |
| ME147 | *amyE:: P_xyl_-gfp-mbl-cat, manA::kan* |  |
| ME155 | *amyE::P_manA_-manA_H97A_-cat* | *amyE::P_manA_-manA_H97A_-cat* allele was constructed using plasmid pME30. |
| ME156 | *amyE::P_manA_-manA_R192A_-cat* | *amyE::P_manA_-manA_R192A_-cat* allele was constructed using plasmid pME31. |
| ME162 | *amyE::P_manA_-manA_H97A_-cat, manA::kan* |  |
| ME163 | *amyE::P_manA_-manA_R192A_-cat, manA::kan* |  |

Strains are derivatives of the wild-type PY79 strain, except for ME136, which is a derivative of the wild-type 168 strain.

* For long-flanking-homology PCR replacement strategy, primer pairs: 198+234, and 232+233 were used to amplify the *manA* flanking genomic regions. Next, the PCR products were used as primers to amplify the *kan* gene from pDG784 [6]. The resultant PCR product was used to transform PY79 strain.

** For long-flanking-homology PCR replacement strategy, primer pairs: 975+976, and 977+978 were used to amplify the *pgi* flanking genomic regions. Next, the PCR products were used as primers to amplify the *erm* gene from pDG646 [6]. The resultant PCR product was used to transform PY79 strain.

**References**

1. Youngman P, Perkins JB, Losick R (1984) Construction of a cloning site near one end of Tn917 into which foreign DNA may be inserted without affecting transposition in Bacillus subtilis or expression of the transposon-borne *erm* gene. Plasmid 12: 1-9.

2. Ben-Yehuda S, Rudner DZ, Losick R (2003) RacA, a bacterial protein that anchors chromosomes to the cell poles. Science 299: 532-536.

3. Wach A (1996) PCR-synthesis of marker cassettes with long flanking homology regions for gene disruptions in *S. cerevisiae*. Yeast 12: 259-265.

4. Sullivan NL, Marquis KA, Rudner DZ (2009) Recruitment of SMC by ParB-*parS* organizes the origin region and promotes efficient chromosome segregation. Cell 137: 697-707.

5. Bejerano-Sagie M, Oppenheimer-Shaanan Y, Berlatzky I, Rouvinski A, Meyerovich M, et al. (2006) A checkpoint protein that scans the chromosome for damage at the start of sporulation in *Bacillus subtilis*. Cell 125: 679-690.

6. Guerout-Fleury AM, Shazand K, Frandsen N, Stragier P (1995) Antibiotic-resistance cassettes for *Bacillus subtilis*. Gene 167: 335-336.
