# Supplementary material for: The Metabolic Enzyme ManA Reveals a Link between Cell Wall Integrity and Chromosome Morphology
Source: PLoS Genet. 2010 Sep 16;6(9):e1001119. doi: 10.1371/journal.pgen.1001119 (PMC2940726; doi:10.1371/journal.pgen.1001119)
Supplement: Text S1 — Supporting materials and methods. (0.04 MB DOC) [file pgen.1001119.s010.doc]

**Text S1**

**Supporting Materials and Methods**

**Plasmid construction**

Plasmid construction was performed in *E. coli* DH5α using standard methods. Primers used for PCR amplifications are listed in Table S1. **pME14**, which contains the *P_manA_* with flanking *amyE* sequences and a chloramphenicol resistance gene (*cat*), was constructed by amplifying the *P_manA_* by PCR using primers 255 and 256. The PCR-amplified DNA was digested with *Bam*HI and *Eco*RI and cloned into the *Bam*HI and *Eco*RI sites of pDG364 (*amyE::cat*) [1]. **pME15** which contains the *manA* gene (promoter and ORF) with flanking *amyE* sequences and a *cat* gene, was constructed by amplifying the *manA* gene by PCR using primers 257 and 258. The PCR-amplified DNA was digested with *Not*I and *Mfe*I and cloned into the *Not*I and *Eco*RI sites of pME14. **pME17**, which contains the 3’ region of *manA* fused to *gfp*, was constructed by amplifying the 3’ region of *manA* gene by PCR using the primers 277 and 278, which replaced the stop codon with *Xho*I site. The PCR-amplified DNA was digested with *Mfe*I and *Xho*I and was cloned into the *Eco*RI and *Xho*I sites of pKL147 (*spc*) [2], which contains the *gfp* coding sequence. **pME25** which contains the 3’ region of *pmi* fused to *gfp*, was constructed by amplifying the 3’ region of *pmi* gene by PCR using the primers 790 and 791, which replaced the stop codon with *Xho*I site. The PCR-amplified DNA was digested with *EcoR*I and *Xho*I and was cloned into the *EcoR*I and *Xho*I sites of pKL147 (*spc*) [2], which contains the *gfp* coding sequence. **pME28** which contains the 3’ region of *tagO* fused to *gfp*, was constructed by amplifying the 3’ region of *tagO* gene by PCR using the primers 985 and 986, which replaced the stop codon with *Xho*I site. The PCR-amplified DNA was digested with *EcoR*I and *Xho*I and was cloned into the *EcoR*I and *Xho*I sites of pKL147 (*spc*) [2], which contains the *gfp* coding sequence. **pME29** which contains the ORF region of *mbl* fused to *gfp* under the control of *P_xyl_* promoter, was constructed by amplifying the ORF of *mbl* by PCR using the primers 987 and 988. The PCR-amplified DNA was digested with *Not*I and *BamH*I and was cloned into the *Not*I and *BamH*I sites of pEA18 (*amyE::cat, P_xyl_*) [3]. **pME30** which contains *P_manA_-manA_H97A_*, whereby amino acid histidine at position 97 of the ManA ORF was replaced with alanine, was constructed by amplifying pME15 (*amyE::manA-cat*) by PCR using the complementary primers 993 and 994 bearing the indicated mutation. Methylated and hemimethylated strands were digested by *Dpn*I. Reactions were carried out according to the QuikChange site-directed mutagenesis protocol (Stratagene). Plasmid was sequenced to verify the presence of the mutation. **pME31** which contains *P_manA_-manA_R192A_*, whereby amino acid arginine at position 192 of the ManA ORF was replaced with alanine, was constructed by amplifying pME15 (*amyE::manA-cat*) by PCR using the complementary primers 995 and 996 bearing the indicated mutation. Methylated and hemimethylated strands were digested by *Dpn*I. Reactions were carried out according to the QuikChange site-directed mutagenesis protocol (Stratagene). Plasmid was sequenced to verify the presence of the mutation.

**References**

1. Harwood CR, Cutting SM (1990) Molecular biological methods for *Bacillus*. Chichester ; New York: Wiley. xxxv, 581 p. p.

2. Lemon KP, Grossman AD (1998) Localization of bacterial DNA polymerase: evidence for a factory model of replication. Science 282: 1516-1519.

3. Gueiros-Filho FJ, Losick R (2002) A widely conserved bacterial cell division protein that promotes assembly of the tubulin-like protein FtsZ. Genes Dev 16: 2544-2556.
